# Lightweight Deep Learning Model for Marketing Strategy Optimization and Characteristic Analysis

**DOI:** 10.1155/2022/2429748

**Published:** 2022-08-23

**Authors:** Yang Su, Chonghong Wang, Xuejiao Sun

**Affiliations:** Yantai Institute of Technology, School of Economics and Management, Yantai 264005, China

## Abstract

The business model of traditional market is declining day by day, and people's consumption cognition has risen to a new level with the leap in science and technology. Enterprises need to adjust and optimize their marketing strategies in time according to the new consumption characteristics, so as to smoothly adapt to the environmental changes in the Internet age. This paper briefly analyzes the relationship between sales development and psychology and constructs a fusion model that can predict preferences with the help of neural network structure of the deep learning method. Describe user portraits and characteristics, analyze users' purchasing behavior and credit literacy, and push related products combined with a hash algorithm to achieve accurate e-commerce marketing purposes. The results show that (1) the model constructed in this paper and five different models are used for multi-modal recognition analysis: the accuracy is 79.56%, the recall rate is 77.43%, F1 is 0.785, and the error value can be reduced to about 0.18 by epoch iteration; the model is superior and has great use value. (2) Using the model to extract user attribute features and predict certain preferences, 13 topics and weight ratios are obtained for users of a certain platform, and the portrait model of each user is constructed. (3) According to the portrait optimization, 8 different marketing strategies are obtained, and the marketing effect is remarkable, fluctuating between 69% and 82%, and the income situation is also satisfactory. The final model design is reasonable and the data performance is good, which provides an intelligent and efficient dynamic strategy service for enterprises.

## 1. Introduction

In the era of information explosion, the way of computer communication technology has changed, and people can complete the whole process of challenging, pricing goods or services without going out on the material platform and interconnected financial service. It is also a big challenge for each attempt to move the market. Consumers have more information channels. They no longer pay more attention to the sales promotion of merchants but pay more attention to the sexual price and sufficient demand for goods themselves. They also tend to pursue new things and expect 24-hour all-round service. Due to the shift of the focus of purchasing characteristics, which drives the shift of marketing focus, enterprises need to cater to the psychology of users and formulate different strategies for different users. However, relying solely on the strength of enterprise decision-making and marketing personnel cannot cope with the massive user data, and it is easy to miss the market outlet and lose the opportunity to expand the reputation and influence of enterprises. Therefore, adhering to the concept of innovation and development, we collect relevant information for reference.

Design a multi-level network structure model, according to the data-driven and key characteristics of the development of gas station marketing strategy, and forecast oil sales [1]. Using the PaaS platform of Kubernetes container to construct TensorFlow container, unified resource scheduling management, and API access control service architecture [2]. Using stuttering word segmentation algorithm, SIFT method, PGBN deep learning model, and Gibbs up-down sampling method, an accurate marketing push is realized [3]. DCNN is used to extract feature vectors, regression analysis method is used to evaluate face value, and personalized products and services are output [4]. Based on the deep learning of Maker Space as the carrier, the marketing major designs experimental teaching [5]. This paper studies the formation mechanism of marketing dynamic capability of international enterprises from the perspective of knowledge [6]. Combined with the characteristics and teaching experience of marketing specialty, this paper explores [7]. Neural marketing based on brain waves, combined with five 3D CNN and multi-layer LSTM prediction models, predicts consumers' preference for products [8]. By establishing a fusion model, artificial feature screening, and tree model for various machine learning algorithms, new ideas are provided for judging users' financial consumption behavior [9]. Test the combination form and effectiveness of graphic information, and explore the influence of consumer behavior and brand relationship [10]. Provide personalized recommendation service for consumers through multi-source big data recommendation system [11]. Using DCNN to learn binary hash coding, large-scale image retrieval is completed in low-dimensional Hamming space [12]. Logistic equations and particle swarm optimization algorithms are introduced to evolve and predict the time series of Internet communication behavior [13]. Based on the three mobile Internet portals of WeChat, Weibo, and APP, this paper studies the optimization of marketing strategy on railway lines [14]. CiteSpace software analyzes business intelligence at home and abroad, and measures management decisions and technology applications [15].

Based on the description of the above literature, this topic has a large number of theoretical basis and experimental ideas that can be used for reference. Change the marketing strategy into online mode, read the text, images, audio, and other contents of the network, and deal with the relevance of dynamic data with the help of business characteristics and users' consumption habits. Describe the user consumption portrait, and build a prediction model with mixed DCNN and LSTM neural network structure. In addition, a recommendation framework based on deep hash is introduced as a supplement, hoping to bring users a better experience and achieve more accurate marketing methods than traditional methods.

## 2. Theoretical Basis

### 2.1. Marketing Strategy

Marketing Strategy [16]; this kind of enterprise activity is mainly organized and operated in a planned way for the market, and obtains sales volume, purchasing power information, and industry prestige value according to various needs and past experience of certain target customers, including comprehensive strategies such as price, promotion, channel, and public relations. Marketing is a means that people, who receive news accept and appreciate the products, services, and benefits introduced and promoted by enterprises. Its main purpose is to make products accepted by consumers, provide customers with satisfactory goods or required services, improve the purchase and use effect, and at the same time establish a brand effect. Because this is a dynamic and changing process, decision-makers need to adjust, create and make plans repeatedly with the changes in the market. Use the 4P principle [17]; according to the actual situation, we should optimize the strategic policy, selectively capture the target market, enhance the competitiveness, and strive to obtain the best economic results with the least input cost in the big environment. Focusing on the seven characteristics of marketing, such as purpose, foresight, uncertainty, systematicness, creativity, debugging, and dynamics, we can carry out effective marketing according to the types and characteristics of different markets.

### 2.2. Overview of Deep Learning

The realization of AI is inseparable from the application of deep learning (DL), which belongs to the extension of machine learning (ML) in AI field. It comes from researchers' exploration of ANN, that is, simulating and approximating biological neural networks. DL can be simply understood as feature learning or representation learning. Through multi-level processing, DL represents features from low level to the high level and completes complex learning tasks such as classification and recognition. Taking the deep learning structure of multi-layer perceptron as an example, it is composed of multiple hidden layers, which can solve the problem of linear indivisibility of a single layer. DL [18] is a general name for a class of pattern analysis methods; it mainly involves CNN [19], Autoencoder [20], and DBN [21]. In recent years, in order to have a deeper understanding of the characteristics and practical applications of DL, researchers have chosen to combine the three methods, which not only increase the parameters but also increase the training difficulty of the model. However, compared with the traditional models, these new fusion models have achieved excellent performance. This paper trains the hybrid network model of DCNN and LSTM to deal with complex transactions.

### 2.3. Deep Convolution Neural Network

Convolution neural network is a kind of feedforward neural network [22]. It is a hierarchical structure, and each convolution layer contains a certain number of convolution kernels. When the convolution layer and the pooling layer interact with each other, the features of the entered data are extracted, the data features are mapped and the output dimension size is reduced. Scholars represented by LeCun designed and trained the classical LeNet-5 model based on the principle of neural cognitive machine and BP algorithm. As the most classical CNN structure, many subsequent works need to improve this model to achieve. Using DCNN of VGGnet series and using multiple nonlinear feature extraction stages to automatically learn hierarchical representation, the computation can be greatly reduced. The operation process of convolution layer convolution is as follows:

(1) $$\begin{array}{c} y_{n}^{l}=f\left(\sum_{\forall m}\left(y_{m}^{l-1}*c_{n,m}^{l}\right)+b_{n}^{l}\right.). \end{array}$$

After complex neuron calculation, DCNN needs to be judged by activation and function, and then can output recognition results, which can improve the recognition ability of neural network. Therefore, our formulas (2)–(4), respectively, introduce the three commonly used activation functions of Sigmoid, ReLu, and tanh, which can solve the problem of gradient disappearance.

(2) $$\begin{array}{c} f\left(x\right)=\frac{1}{1+e^{-x}}, \end{array}$$

(3) $$\begin{array}{c} \text{ReLu}\left(x\right)=\text{max}\left(0,x\right), \end{array}$$

(4) $$\begin{array}{c} f\left(x\right)=\frac{e^{x}-e^{-x}}{e^{x}+e^{-x}}. \end{array}$$

The calculation form of convolution layer is introduced.

(5) $$\begin{array}{c} y_{j}^{l}=f\left(z_{j}^{l}\right),\\ z_{j}^{l}=\sum_{\forall m}y_{i}^{l-1}*c_{ij}^{l}+b_{j}^{l}. \end{array}$$

Weights [23] and thresholds [24] are obtained by random gradient descent method.

(6) $$\begin{array}{c} c_{ij}^{l}=c_{ij}^{l}+\alpha \frac{\partial L\left(c_{ij}^{l}\right)}{\partial c_{ij}^{l}},\\ b_{j}^{l}=b_{j}^{l}+\alpha \frac{\partial L\left(b_{j}^{l}\right)}{\partial b_{j}^{l}}. \end{array}$$

### 2.4. Long-Term and Short-Term Memory Neural Network

LSTM model is a special RNN [25]. It makes some changes in the neuron structure of RNN and introduces memory units to solve the problem of gradient explosion and disappearance that traditional RNN cannot avoid. Gradient learning algorithm is introduced into the network structure of LSTM, and a processor is added to judge whether information is useful or not, which includes three gates: input, forgetting, and output. In addition, it also has a memory unit that can improve the ability to process long sequence data. First of all, LSTM needs to decide the information to be discarded in the cell state, and view *h*_*t*−1_¸*x*_*t*_ through the forgetting gate to output the 0–1 vector, as shown in Formula (7). Then the information of the cell state is updated by the input gate and tanh to obtain Formulas (8) and (9). After updating the new unit information *C*_*t*_, the output state characteristics are judged, and the vectors between [−1, 1] are obtained by sigmoid and tanh. Finally, the RNN unit is output, and the vector is multiplied by the judgment condition to obtain a Formulas (12) and (11).

(7) $$\begin{array}{c} f_{t}=\sigma \left(W_{f}•\left[h_{t-1},x_{t}\right]+b_{f}\right), \end{array}$$

(8) $$\begin{array}{c} i_{t}=\sigma \left(W_{i}•\left[h_{t-1},x_{t}\right]+b_{i}\right), \end{array}$$

(9) $$\begin{array}{c} C_{t}=\tanh\left(W_{c}•\left[h_{t-1},x_{t}\right]+b_{c}\right), \end{array}$$

(10) $$\begin{array}{c} o_{t}=\sigma \left(W_{o}\left[h_{t-1},x_{t}\right]+b_{o}\right), \end{array}$$

(11) $$\begin{array}{c} h_{t}=o_{t}*\;\tanh\left(C_{t}\right). \end{array}$$

Attention mechanism can solve the capacity problem and long-term dependence problem of coding vector, effectively shorten the distance of information transmission and reduce unnecessary steps. So, the LSTM model is optimized. After input *x*={*x*_1_, *x*_2_,…, *x*_*n*_}, the attention mechanism is introduced when the hidden layer outputs vector *h*. According to the encoder output and attention probability, the average value is calculated after addition. Where *A*_*i*_ stands for the probability of attention; matrix parameters are represented by *w*, *W*, *U*; *b* is the biased vector; tanh is a hyperbolic tangent function.

After introducing the attention mechanism, the related calculations are as follows:

(12) $$\begin{array}{c} A_{i}=\frac{\text{exp}\left(f\left(\bar{h},h_{i}\right)\right)}{\sum_{j}\text{exp}\left(f\left(\bar{h},h_{j}\right)\right)},\\ f\left(\bar{h},h_{i}\right)=w^{T}\tanh\left(W\bar{h}+Uh_{i}+b\right),\\ V=\sum_{i=0}^{t}A_{i}h_{i}. \end{array}$$

### 2.5. Binary Hash Algorithm

A binary hash layer is introduced to construct an independent hash function. When the feature passes through this layer, the hash code can be obtained. Then, the hash code passes through the loss layer to obtain the optimized parameters calculated by the loss function. Hash codes can be expressed as

(13) $$\begin{array}{c} {\left(h_{1},h_{2},\ldots ,h_{q}\right)}^{T}={\left(\text{sign}\left(Wx\right)\right)}^{T}. \end{array}$$

Sub features that can enter the full connection layer are obtained from the slice layer.

(14) $$\begin{array}{c} f_{i}\left(x^{\left(i\right)}\right)=W_{i}x^{\left(i\right)},i=1,2,\ldots ,q. \end{array}$$

The activation layer maps the one-dimensional value output by each block.

(15) $$\begin{array}{c} \tanh\left(v^{\left(i\right)}\right)=\frac{1-c^{\beta v^{\left(i\right)}}}{1+c^{\beta v^{\left(i\right)}}},i=1,2,\ldots ,q,\\ v^{\left(i\right)}=f_{i}\left(x^{\left(i\right)}\right). \end{array}$$

Enter the merging layer and represent vectors.

(16) $$\begin{array}{c} s={\left(v^{\left(1\right)},v^{\left(2\right)},\ldots ,v^{\left(q\right)}\right)}^{T}. \end{array}$$

Enter the thresholding layer.

(17) $$\begin{array}{c} g\left(s^{\left(i\right)}\right)=\left\{\begin{array}{cc} 1, & s^{\left(i\right)}\geq 0,\\ -1, & s^{\left(i\right)}<0. \end{array}\right) \end{array}$$

The quantization error loss is added to the objective function.

(18) $$\begin{array}{c} L_{q}=\frac{1}{2}{\left\|h-s\right\|}_{2}^{2}. \end{array}$$

Combined with SoftMax, the overall loss function is obtained.

(19) $$\begin{array}{c} L_{T}=L_{s}+\lambda L_{q}. \end{array}$$

## 3. Design and Implementation of 3 System Model

### 3.1. User Portrait Model

The characteristics of the times make more and more people active on the Internet. People use but are not limited to words, pictures, videos, and other ways to record their lives and work in this online world and express their preferences freely and fully. Social activity data on the Internet will record a person's interests, behavior habits, psychological state, and other personal attributes. Major small and medium-sized enterprises should seize the opportunity, open up their thinking, and realize accurate marketing according to the distinctive personal characteristics of Internet users. Adjust the original strategy, carry out personalized analysis and feature optimization of products, and mine the value hidden in the data.

In view of the universality and practicability of the user portrait model, this paper also adds this mining method, which classifies users and labels them according to the features extracted from the DL model, so as to facilitate more detailed consumption analysis. First of all, we need to collect the user's basic data, and then preprocess the user's text data source and picture data source. Finally, the attributes are abstracted by a genetic algorithm, Bayesian algorithm, and neural network algorithm, and the useable user portrait is successfully established.

The hierarchical structure of portrait construction is as follows:

(20) $$\begin{array}{c} \left\{\begin{array}{c} \Theta_{j}^{\left(T\right)}:\text{Gam}\left(r,\left(\frac{1}{c_{j}^{\left(T+1\right)}}\right)\right),\\ \Theta_{j}^{\left(T\right)}:\text{Gam}\left(\left[\begin{array}{c} \phi_{w}^{\left(t+1\right)}\phi_{w-j}^{\left(t+1\right)}\\ \phi_{v}^{\left(t+1\right)}\phi_{v-j}^{\left(t+1\right)} \end{array}\right],\left(\frac{1}{c_{j}^{\left(t+1\right)}}\right)\right),\\ \Theta_{j}^{\left(1\right)}:\text{Gam}\left(\left[\begin{array}{c} \phi_{w}^{\left(2\right)}\phi_{w-j}^{\left(2\right)}\\ \phi_{v}^{\left(2\right)}\phi_{v-j}^{\left(2\right)} \end{array}\right],\left(\frac{p_{j}^{\left(2\right)}}{\left(1-p_{j}^{\left(2\right)}\right)}\right)\right). \end{array}\right) \end{array}$$

After simplification and derivation, we can get the following formula:

(21) $$\begin{array}{c} \left\{\begin{array}{c} w_{j}^{\left(t\right)}:\text{Pois}\left(-\phi_{w}^{\left(t\right)}\theta_{w-j}^{\left(t\right)},\text{ln}\left(1-p_{j}^{\left(t\right)}\right)\right),\\ v_{j}^{\left(t\right)}:\text{Pois}\left(-\phi_{v}^{\left(t\right)}\theta_{v-j}^{\left(t\right)},\text{ln}\left(1-p_{j}^{\left(t\right)}\right)\right). \end{array}\right) \end{array}$$

### 3.2. Preference Recommendation and Strategy Adjustment

Traditional marketing methods cannot meet the needs of Internet users, and cannot play a very good role in publicity, but greatly increase costs, while achieving little effect. In order to improve the loyalty and satisfaction of users and achieve the predetermined sales volume and reputation, recommending the relevant preferences of users is the key. The preference here means that users have a good impression of specific goods or services, and are more inclined to buy trusted brands or trademark products when consuming, that is, consumption preference. Such users will buy repeatedly under the influence of subconscious habits. In addition, users are no longer limited to ordinary and popular standard goods after having a variety of choices. They are easily influenced by their own psychology, environment, and culture, and have a strong willingness to choose products that can satisfy their individualized pursuit. The previous experimental design has laid the foundation for our preference recommendation. We can analyze the user's love and habit of a certain kind of product or a certain kind of service, and turn this market information that can be mastered into the core of marketing strategy.

1. For consumers, who care about the price and practicality of goods or services, take affordable promotion as the marketing strategy, and push full reduction activities, coupons, and discounted goods for these people. They cannot control their desire to buy, and they cannot help buying more goods or services.
2. Not everyone measures the value of goods by cost performance, and there are also main buyers who, pay more attention to new things. They do not care about the amount of money, but pay attention to the trend of the times, the style and novelty of goods. They can pay generously for a service that has only become popular in society, just to satisfy the novelty of the moment; you can also snap up a small commodity because of its limited sales. Therefore, aiming at this part of consumers, we should constantly update and focus on the marketing of new models and early adopters.
3. For people, who go to work and study, due to the limitation of time and space, they cannot participate in offline shopping experience activities anytime and anywhere. They need convenient, fast and simple shopping choices, and can enjoy centralized customer service and after-sales service at any time.

### 3.3. Framework of Hybrid Model

**Figure 1:**
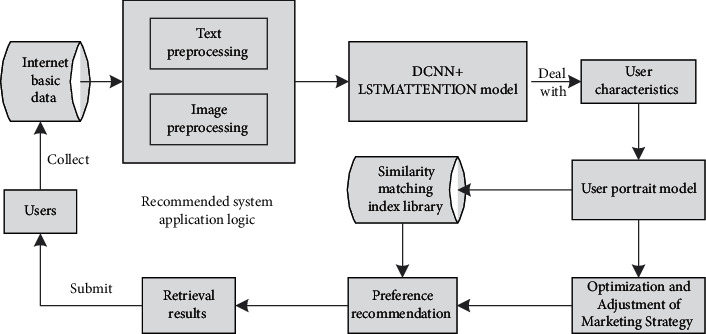
Schematic diagram of DL-based hybrid model.

The model of this paper is based on DCNN and LSTM neural network with an attention mechanism, which also introduces a binary hash layer so that the overall processing ability of the model is higher. According to the performance of users on the Internet, collect basic data, then preprocess the text and pictures, and transmit them to DCNN + LSTMATTENTION model to find various hidden user characteristics. According to the information characteristics of these users, the user portrait model is constructed, and the marketing strategy is optimized and adjusted. In order to better cater to consumers' ideas, analyze decision-making behavior, search the index database of similarity matching, recommend preferred products that meet consumers' interests, and make the adjusted marketing strategy play a role. Finally, the retrieval results are submitted to users for use, which directly affects the transactions of consumer customers as shown in Figure 1.

## 4. Experimental Analysis

### 4.1. Performance Results of the Model

**Figure 2:**
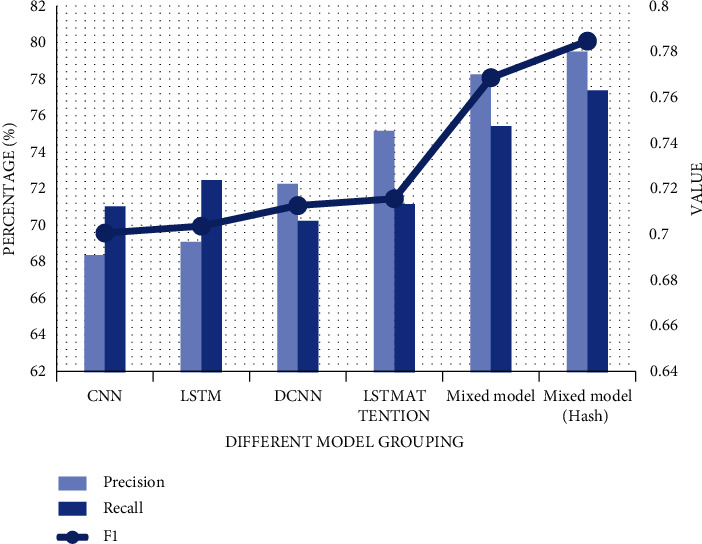
Comparison of multi-modal recognition results.

In this section of the experiment, we will explain the reasons for choosing the hybrid model. By identifying and detecting the graphic text of consumer shopping, we record the three indicators identified by multimodality respectively. In order to highlight the superiority of this model, CNN, LSTM, DCNN, LSTMATTENTION, basic hybrid model (DCNN + LSTMATTENTION), and mature hybrid model with hash layer are compared. The recognition accuracy of CNN model is 68.42%, the recall rate is 71.08%, and the F1 score is about 0.701. LSTM and its value fluctuate between 1% and 2%, and their performance is roughly the same. The performance of DCNN and LSTMATTENTION models is slightly better than the first two models, with accuracy of 72.31% and 75.22%, respectively, and there is still room for optimization and improvement. The data of the common hybrid model performs well. After adding Hash, the accuracy of the fusion model is as high as 79.56%, the recall rate rises to 77.43%, and the equilibrium F score reaches 0.785. The larger the value, the better the model as shown in Figure 2.

**Figure 3:**
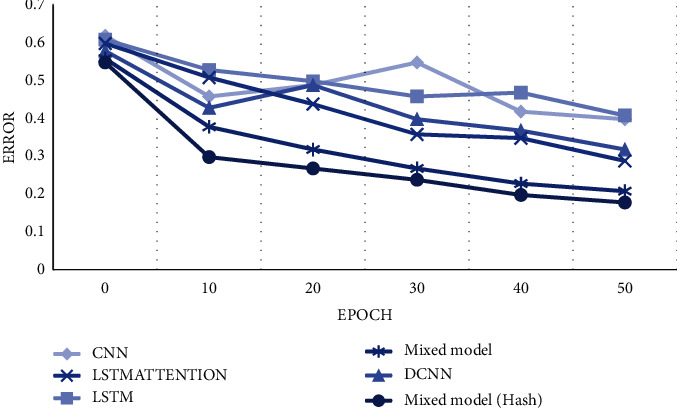
Comparative analysis of errors.

After testing the above indexes, the error values of each model are analyzed. With the increase of the number of an epoch, the weight of the model is set to undergo repeated update iterative training. With the increase of epoch, their diversity becomes stronger and stronger, and the curves transition from the initial nonfitting state to the fitting state. The method in this paper not only reduces the weight parameters but also reduces the prediction error of the model. After 50 updates, its error value can be reduced to about 0.18. Compared with other models, the error value of this model does not appear high or low, and its dynamic performance is obviously better than other basic network models. Finally, we can find that the performance of the model designed by this method is better than that of other single models and common mixed models, and the effect is satisfactory as shown in Figure 3.

### 4.2. Preference Prediction Analysis

**Figure 4:**
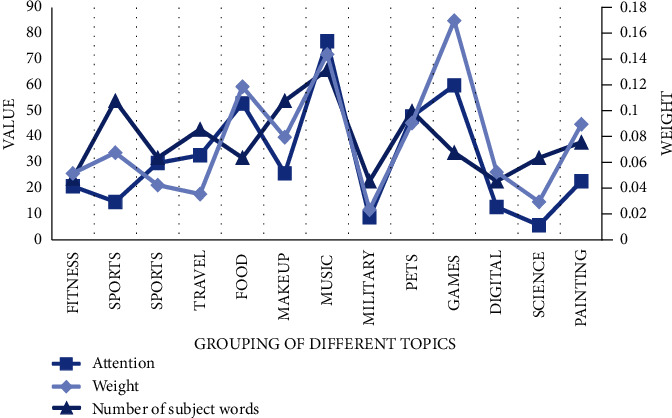
Weight information for part of the topic.

First of all, we collect the basic data of users' Internet, and after processing the model, we can determine the topic content and its weight bias that users prefer. Most subject words under each topic will have different characteristics, which are identifiable and unique, and can well distinguish the personal attributes of different users from their corresponding hobbies. Confirming these themes can better assist the construction of users' portraits and strive to create a database of one person and one portrait. A total of 1000 active users of a certain platform were randomly selected, the data containing text pictures were crawled as the original data, and 13 topics with a high topic degree were obtained, each topic had 20∼70 subject words as shown in Figure 4.

**Figure 5:**
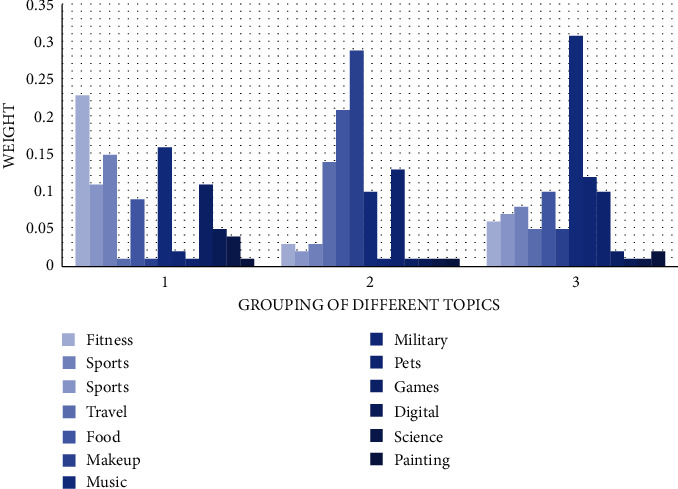
Preference prediction of user portrait.

Three users who volunteered to participate in the experimental test were selected, the training data set was processed and constructed, and their different preferences were predicted by portraits. It is obvious that they have different tendency distribution in theme preference, which proves that they pay close attention to and have a strong interest in a certain kind or several themes in daily life. We can clearly see that the first user prefers topics such as physical fitness, followed by music. The second user focused on travel, food, and beauty, and was interested in music and pets. The last user is interested in music, with a weight ratio as high as 31%, and pays attention to food, military affairs, and pets as shown in Figure 5.

### 4.3. Analysis of Marketing Strategy and Recommendation Effect

**Figure 6:**
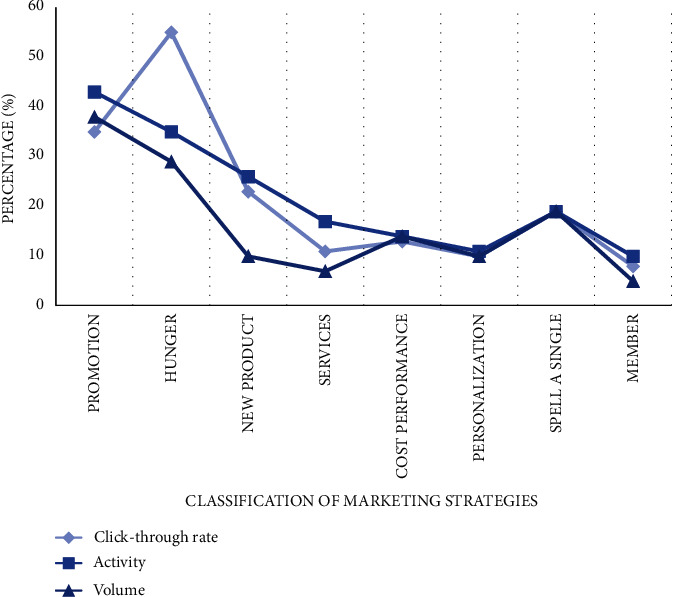
Marketing strategy optimization.

After using network and model to extract users' attributes effectively, enterprises can adjust different markets and optimize marketing strategies for different types of users. We analyzed and counted the increased percentage points of 8 adjusted marketing strategies, and compared the click-through rate, activity, and turnover of goods or services with the data of traditional marketing models. From the curve trend in the figure, we can find that preferential promotion and hunger marketing have the best effect, and the click-through rate and activity have increased by more than 20%. Secondly, the new product launch and group purchase/spelling strategy bring considerable benefits. Other strategies have increased to varying degrees, which proves that the adjusted and optimized model is very practical as shown in Figure 6.

**Figure 7:**
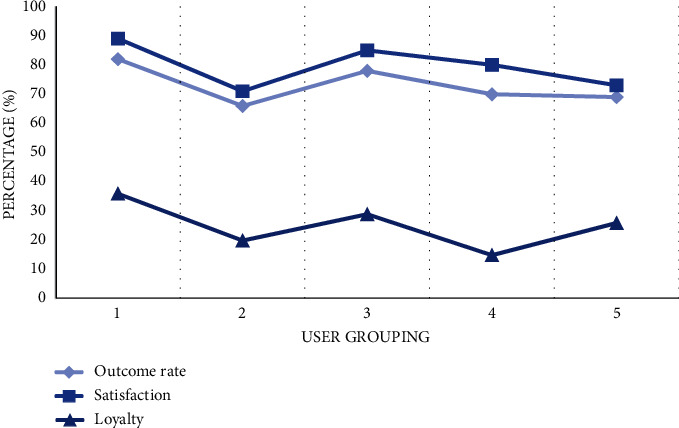
Recommended effect feedback.

After adjustment, this section also needs to evaluate the effect of the optimized strategy. Recycling five groups of users (10 people in each group) in the marketing achievement rate, consumer satisfaction, and brand loyalty of these three indicators of evaluation. According to these data, we can evaluate the actual effect and true value of the model after use, so as to carry out feedback and correction work. Because of different marketing strategies and individual differences, the index value of each group fluctuates to a certain extent. From the perspective of marketing results, the values of the five groups are between 69% and 82%, which has good results; consumer satisfaction is between 70% and 90%. However, brand loyalty needs long-term accumulation of word of mouth, so it is generally low, which needs further testing and research as shown in Figure 7.

## 5. Conclusion

Traditional marketing relies on the investment of manpower and capital and needs a large number of advertisements to put into the market, so as to arouse customers' desire to buy, establish the image of products, and make them deeply rooted in the hearts of the people. Customers can only choose the products and services provided by merchants or enterprises within a certain range, ignoring their different feelings. However, due to the rise of e-commerce, consumers are more willing to choose products with personality signs, low cost and good quality, and guaranteed after-sales service. Traditional marketing strategy in this online shopping trend does not have much effect, enterprises need an optimization model that can constantly adjust marketing strategy. Based on this research background, the experiment in this paper takes the Internet as the basic business environment and enriches the field of personalized recommendation from all aspects and angles. Combining the purpose of the previous sale with the existing demand, we will promote e-commerce marketing that is accurate to everyone. Customers can enjoy high-quality services and fully meet individual needs. Enterprises also exceed their marketing objectives while expanding their influence, which has certain practical value. In this paper, combined with multi-modal data, the experiment based on a deep learning model can be done, and the completion and accuracy of the results are satisfactory. Because the research of deep learning is still in the development stage, there are many places that need to be further revised and improved in this experiment. In addition to the challenges faced by the fusion model algorithm, future research work should focus on the dynamic development process of marketing strategy facing the market. At present, the push function of marketing is relatively simple, which is only for the products that users are interested in. It can also appropriately enhance the broadcasting of online advertisements and send a variety of preferential policies for promotions and discounts to attract more customers. There is still a lack of inspection and analysis of overall operation defects, so as to ensure the normal operation of equipment in actual deployment. More data sets and features are still needed for testing, and the variability of parameters in different scenarios is worth exploring.

## Data Availability

The experimental data used to support the findings of this study are available from the corresponding author upon request.
